# Conformal mapping for multiple terminals

**DOI:** 10.1038/srep36918

**Published:** 2016-11-10

**Authors:** Weimin Wang, Wenying Ma, Qiang Wang, Hao Ren

**Affiliations:** 1 State Key Laboratory of Optical Technologies on Nano-Fabrication and Micro-Engineering, Institute of Optics and Electronics, Chinese Academy of Sciences, Chengdu 610209, China; 2 College of Communication Engineering, Chengdu University of Information Technology, Chengdu 610225, China; 3 School of Electrical, Computer, and Energy Engineering, Arizona State University, Tempe, Arizona 85281, USA

## Abstract

Conformal mapping is an important mathematical tool that can be used to solve various physical and engineering problems in many fields, including electrostatics, fluid mechanics, classical mechanics, and transformation optics. It is an accurate and convenient way to solve problems involving two terminals. However, when faced with problems involving three or more terminals, which are more common in practical applications, existing conformal mapping methods apply assumptions or approximations. A general exact method does not exist for a structure with an arbitrary number of terminals. This study presents a conformal mapping method for multiple terminals. Through an accurate analysis of boundary conditions, additional terminals or boundaries are folded into the inner part of a mapped region. The method is applied to several typical situations, and the calculation process is described for two examples of an electrostatic actuator with three electrodes and of a light beam splitter with three ports. Compared with previously reported results, the solutions for the two examples based on our method are more precise and general. The proposed method is helpful in promoting the application of conformal mapping in analysis of practical problems.

Conformal mapping is one of the most powerful tools of complex analysis, and has been applied in many mathematical and physical fields, including those dealing with transmission lines^1^,^2^,^3^,^4^,^5^, integrated circuit components^6^,^7^,^8^,^9^,^10^,^11^, electrostatic actuators^12^,^13^,^14^,^15^,^16^, transformation optics^17^,^18^,^19^,^20^,^21^, channel flows^22^,^23^, and rough surfaces^24^,^25^,^26^. Conformal mapping transforms a structure with a complex shape into a geometry that makes the problem more easily solvable. In the process of transformation, both the shapes and the sizes of curves in the transformed structure can greatly differ from those in the original structure; however, the angle between any two curves is preserved in the process.

Figure 1 shows an example of the application of the conformal mapping method to electrostatics. The thick solid lines AB and CD represent two conductors and thus they are two equipotential lines. The thin solid lines between AB and CD represent other equipotential lines, and the dashed lines are electric field lines. Because of the inherent relationship of equipotential lines and field lines, solid lines and dashed lines form an orthogonal curvilinear coordinate grid. Through conformal mapping, the area enclosed by curves ABCD (the shaded region in Fig. 1a), is mapped onto a rectangle, as shown in Fig. 1b. All of the mapped field lines and equipotential lines become straight lines and they remain orthogonal because of the preservation of angles. Therefore, those lines form a Cartesian coordinate grid, and the mapped structure represents a perfect (fringe effect-free) parallel plate capacitor whose electric field distribution is widely understood. So, the capacitance, potential, field, and charge of the shaded region can be obtained through inverse mapping. In addition to the parallel plate capacitor, the problem to be solved can also be mapped onto other structures, such as those shown in Fig. 1c,d.

As shown in Fig. 1, each mapped structure comprises only two solid edges. These edges represent the two terminals of the analyzed physical model, which can represent conductors for electrostatics, inlets or outlets for fluid flow, or ports for a light waveguide. However, real structures commonly comprise three or more terminals: the substrates of most microelectronic devices can serve as additional conductors; fluid flow often comprises multiple inlets and outlets; light dividers or couplers have multiple ports. To analyze these structures using the conformal mapping method, multiple terminals are often approximated as two terminals. Taking a structure with three terminals as an example, if two terminals have the same potential, they can be approximately treated as one terminal; so the three terminals are reduced to two terminals^14^,^27^,^28^. If all three terminals are of totally different potentials, the one with the medium potential and the one with a potential closest to it are approximated as one terminal; so again the three terminals are reduced to two terminals^15^,^16^,^29^. In some studies, additional terminals were neglected altogether^14^,^30^. These approximations can introduce errors, the sizes of which are dependent on the shapes and sizes of the analyzed structures. In other words, for structures with multiple terminals, application of the conformal mapping method is severely limited.

In this study, a strict and exact conformal mapping method for multiple terminals is proposed and described in detail. To illustrate the effectiveness and accuracy of the method, two application examples are given, and the calculation results are compared with those in the literature. For clarity, the existing conformal mapping method whose mapped structure comprises only two terminals is called the traditional conformal mapping method to distinguish it from the proposed method.

## Mapping Method

As an example, the proposed method is applied to an electrostatic problem, where terminals generally represent conductors. A structure with two conductors AB and CD of different potentials *V*_1_ and *V*_2_ can be easily mapped using the traditional conformal mapping method. For a structure with an additional conductor (named EF), there are two possible electric configurations for EF: Configuration I with a fixed potential and Configuration II with a fixed net free charge.

In the simplest situation of Configuration I, the fixed potential of the additional conductor EF is *V*_1_ or *V*_2_, i.e., the potential is the same as that of conductor AB or CD. Figure 2a shows a schematic of this situation, where the shaded region is enclosed by three conductors AB, CD and EF, and three field lines BC, DE and FA. Without loss of generality, we assume that conductors EF and CD have the potential *V*_2_ and that conductor AB has the potential *V*_1_ (where *V*_2_ > *V*_1_). Accordingly, the directions of the electric field lines BC and FA are from high potential to low potential, as indicated by the solid arrows in Fig. 2a. With regards to field line DE, as the vertices D and E have the same potential (*V*_2_), the potential from vertices D to E along the dashed line DE cannot change monotonically. The only possibility is that it first decreases and then increases, as symbolized by the hollow arrows in Fig. 2a. So, the potential along the line DE has a local minimum at a point called D’. Once conformally mapped, the only mapped structure of the structure shown in Fig. 2a should resemble that shown in Fig. 2b. There are two reasons for this: (1) In the mapped perfect parallel plate capacitor, electric field line DD’E should be perpendicular to equipotential lines CD and EF; (2) In the mapped capacitor, equipotential lines CD and EF are parallel. As CD has the same potential as EF, they should be collinear. Accordingly, DD’E is folded in D’, and DD’ and D’E are superposed (they are drawn separately in the figure for the sake of clarity). To construct the mapping function from Fig. 2a,b, the position of D’ is the only unknown and can be determined thanks to the fact that the length of DD’ and of D’E must be equal. Once the mapping is constructed, the mapped structure of the original structure with three terminals remains a parallel plate capacitor. Given that the potential and charge are invariant under conformal mapping, the electric field distribution of the mapped capacitor can be easily obtained. Therefore, the analyses are identical to those of structures with two terminals.

In the simplest situation of Configuration II, the additional conductor EF is floating with a net charge of zero. Figure 2c shows a schematic of this situation. Because of the influence of the electric field between conductors AB and CD, inductive charges emerge on EF. As a result, EF must have a potential *V*_3_ satisfying the relationship *V*_2_ > *V*_3_ > *V*_1_. The sum of the positive and negative inductive charges should be zero to ensure the zero net charge of EF. A point E’ denoting the turning point between the positive and the negative inductive charges emerges on EF. In a parallel plate capacitor, all conductors have the same charge densities. Hence, the mapped structure can be drawn in the form shown in Fig. 2d. On the one hand, the charge of EE’ has an opposite polarity to the charge of E’F. As a result, the length of EE’ in the mapped structure must be equal to that of E’F. On the other hand, the equipotential line EF is located in parallel between AB and CD for that *V*_2_ > *V*_3_ > *V*_1_. The position of E’ and the potential of EF can be solved through the superposition of E and F.

A more common situation for Configuration I is the case where the potentials of the three conductors are different to each other. We assume that the additional conductor EF has a potential *V*_3_, where *V*_2_ > *V*_3_ > *V*_1_. Given the different structural parameters, there are two possible electric field distributions, as shown in Fig. 2e,g. In Fig. 2e, all electric field lines start from CD or EF, and terminate at AB, while there are no electric field lines between CD and EF. This field distribution is similar to that shown in Fig. 2a. Under the relationship *V*_2_ > *V*_3_, the mapped structure is shown in Fig. 2f. In Fig. 2g, the electric field lines start from CD, and terminate at both AB and EF. This distribution is similar to that shown in Fig. 2c. Because the potential of EF is fixed and its net charge is unknown, the mapped length of EE’ is usually different to that of E’F, as shown in Fig. 2h. Figure 2f,h shows structures that contain two perfect capacitors with different heights. These two capacitors have the same electric field strength and their potential differences are *V*_3_ − *V*_1_ and *V*_2_ − *V*_1_, respectively. Hence, the position of D’ (Fig. 2f) or E’ (Fig. 2h) can be computed through the relationship (*V*_3_ − *V*_1_)/*L*_FA_ = (*V*_2_ − *V*_1_)/*L*_BC_, where *L* is the length between corresponding vertices. Because of the uniqueness of the solution of the electrostatic boundary-value problem, only one point of D’ and E’ can satisfy this relationship. Once the point is obtained, the corresponding distribution and mapped structure can be achieved.

In the general situation of Configuration II, the conductor EF is floating with a non-zero net charge *Q*. Similar to the zero net free charge situation, EF will have an unknown potential *V*_3_, where *V*_2_ > *V*_3_ > *V*_1_, because of electrostatic induction. The electric field distribution is identical to the situation shown in Fig. 2e,g; so, the mapped structures are as shown in Fig. 2f,h. Similarly, the electric field strength for the right capacitor is (*V*_2_ − *V*_1_)/*L*_BC_ (Fig. 2f,h), while for the left capacitor it is *Q*/(*ε*_0_*ε*_*r*_*H*   *L*_EF_) (Fig. 2f) or *Q*/[*ε*_0_*ε*_*r*_*H*   (*L*_E’F_ − *L*_EE’_)] (Fig. 2h), where *ε*_*r*_ denotes the permittivity of vacuum, *ε*_*r*_ represents the relative permittivity of the shaded region, and *H* is the height of EF along the direction orthogonal to the plane of the figure. Hence the position of D’ or E’ can be determined by


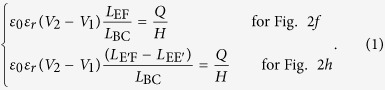


In addition to Configurations I and II, there is another configuration that is characterized by three floating conductors with specific charges. Assuming that the charges of conductors AB, CD, and EF are *Q*_1_, *Q*_2_, and −(*Q*_1_ + *Q*_2_), respectively, their electric field distribution can also be described as that shown in Fig. 2e,g, and therefore, the mapped structure is also the same as that shown in Fig. 2f,h. The position of D’ or E’ is determined by


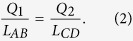


The above analysis shows all possible configurations for three conductors. If a structure comprises more than three conductors, the mapped structure can also be drawn in a similar manner. Assuming that the number of conductors is N, N–2 edges can be folded into the inner section of the mapped rectangle. Figure 2i shows an example of a mapped result for four conductors. The positions of D’ and G’ are determined by a system of quadratic equations with two unknowns.

**Figure 1:**
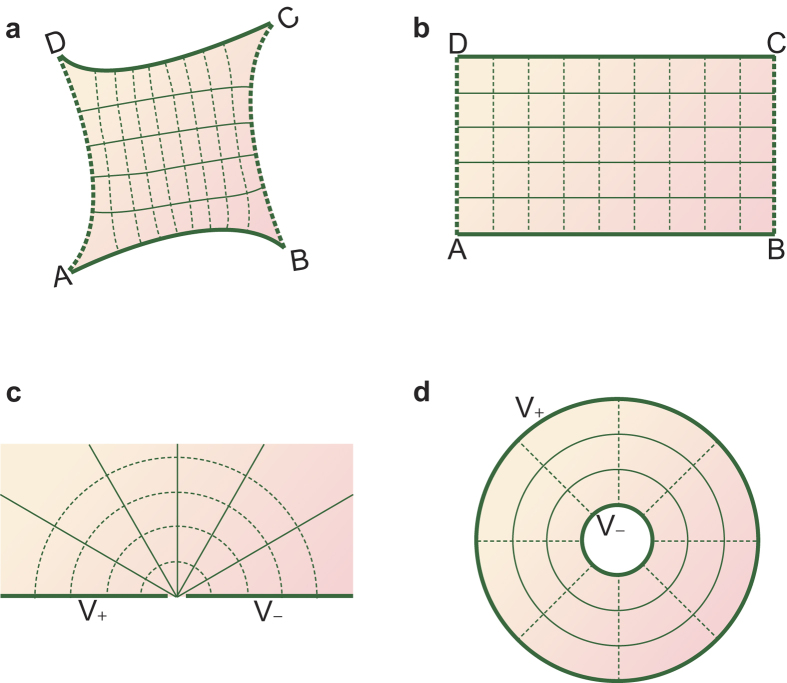
A schematic diagram showing an example of the use of conformal mapping in electrostatics. (**a**) Schematic of the analyzed structure. The thick solid lines are conductors, the thin solid lines are equipotential lines, and the dashed lines are electric field lines. The solid lines and dashed lines form an orthogonal curvilinear coordinate grid. (**b**) Mapped structure of **a**. All mapped solid lines and dashed lines are straight, and they are orthogonal because of the preservation of angles, so they form a Cartesian coordinate grid. (**c**,**d**) Other possible mapped structures. On (**c**) the negative real axis has a potential of *V*_+_, and the positive real axis has a potential of *V*_*−*_. Thus, the concentric circles whose center is the origin are electric field lines, and the straight lines through the origin represent equipotential lines. On (**d**) the electric field lines and equipotential lines are opposite to those of (**c**).

**Figure 2:**
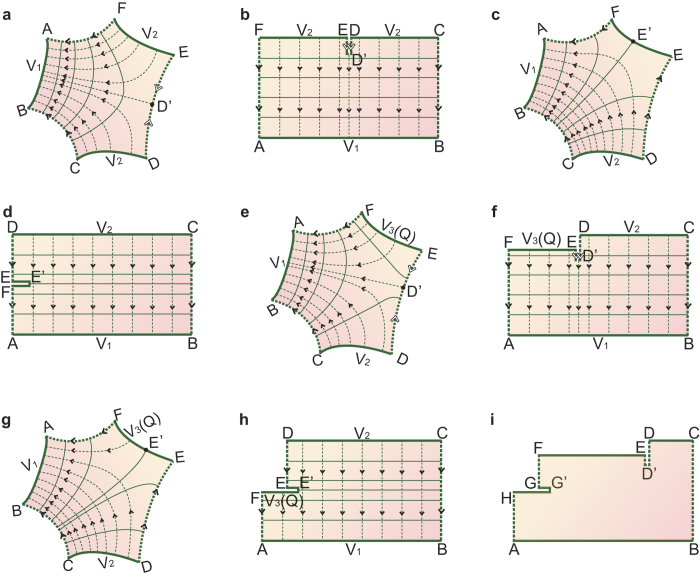
Conformal mapping for multiple conductors. (**a**) Schematic of the simplest situation for Configuration I. The additional conductor EF has the same potential as CD. The directions of the electric field lines BC, DE, and FA are indicated by the arrows shown. (**b**) Mapped structure of (**a**). (**c**) Schematic of the simplest situation for Configuration II. The additional conductor EF is floating and has a zero net free charge. (**d**) Mapped structure of (**c**). (**e**) The first possible electric field distribution for the general situation of Configurations I and II. The potential of the additional conductor EF is different from that of the other two conductors, or it has a non-zero net free charge *Q*. (**f**) Mapped structure of (**e)**. (**g**) Other possible electric field distribution of situation (**e**). (**h**) Mapped structure of **g**. (**i**) One possible conformal mapping result for four conductors, AB, CD, EF, and GH.

In the above configurations, structures with multiple terminals are all mapped onto a rectangle, with the additional terminals folded into its inner part. In this way, these configurations are extensions of Fig. 1b. Moreover, according to the Riemann mapping theorem^31^, any simply connected region, such as the structure shown in Fig. 1c, can also be mapped conformally onto a rectangle.

**Figure 3:**
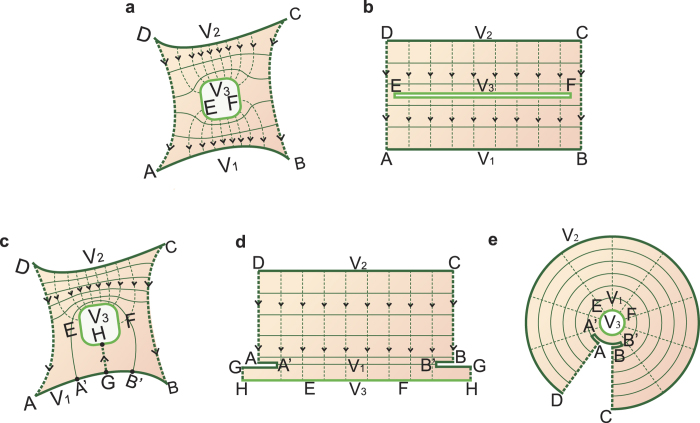
Conformal mapping method for a multiply connected region. (**a**) Schematic of a doubly connected region. A conductor EF is enclosed by the structure shown in Fig. 1a, and its potential satisfies *V*_2_ > *V*_3_ > *V*_1_. (**b**) The conformal mapping structure of **a**. The conductor EF is an infinitely thin closed straight line. (**c**) Another situation, *V*_2_ > *V*_1_ > *V*_3_, for the structure shown in **a**. (**d**) The conformal mapping structure of **c** where the position of an electric field line GH is known. The known field line GH is mapped onto two separate lines. (**e**) The conformal mapping structure of **c** where GH is unknown. It is a circular slit structure, where AB is an infinitely thin folded arc.

For a multiply connected region, the analyses are more complex. Some approaches to derive the mapping function have already been proposed for some multiply connected regions^32^,^33^,^34^,^35^. Here, we describe a general method to determine the appropriate mapped structure for several typical situations of multiply connected region with several terminals. Figure 3a shows a doubly connected structure with three conductors whose potentials are *V*_1_, *V*_2_ and *V*_3_, respectively. The field distribution shown in Fig. 3a represents the situation *V*_2_ > *V*_3_ > *V*_1_ and it can be mapped onto the structure shown in Fig. 3b. For the situation *V*_2_ > *V*_1_ > *V*_3_, one possible field distribution is shown in Fig. 3c. To obtain its mapped structure, the simplest method is to reduce the connectivity of the region through consideration of its symmetry or other restrictions. For example, if the position of an electric field line GH can be obtained on the basis of a certain symmetry, the connectivity of Fig. 3c will decrease from 2 to 1 and it can be mapped onto a rectangle; in this case, vertices G and H are mapped onto two points, respectively, as shown in Fig. 3d. Alternatively, if GH is unknown, Fig. 3c can be mapped onto a folded circular slit structure, as shown in Fig. 3e. Its field and equipotential lines form a polar coordinate grid.

In summary, a conformal mapping method to solve problems of multiple terminals in arbitrary situations is proposed by carefully analyzing boundary conditions. To demonstrate the detailed calculation steps, two examples are given in the following section.

## Application examples

### MEMS electrostatic actuator

**Figure 4:**
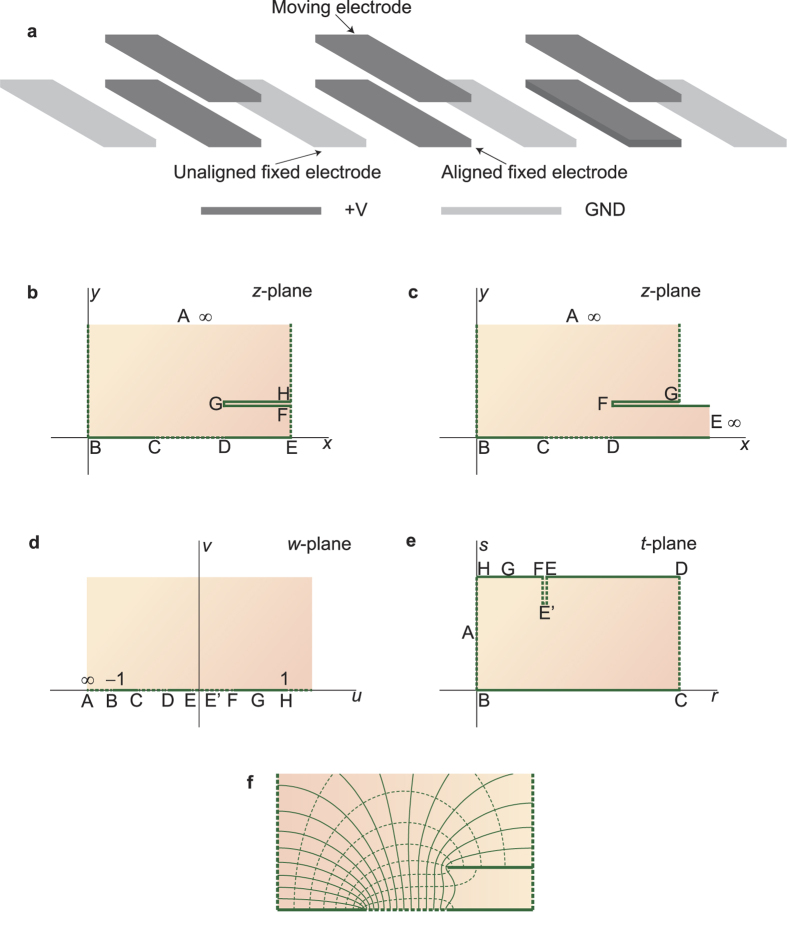
Actuator proposed in ref. 14. (**a**) Schematic diagram of the electrostatic repulsive actuator. (**b**) Modeled structure of the actuator in the *z*-plane, where *z* = *x* + *iy*. (**c**) Simplified structure in ref. 14. (**d**) Structure (**b**) mapped conformally onto the upper half-plane of the *w*-plane, where *w* = *u* + *iv*. (**e**,**d**) is further mapped onto a parallel plate capacitor in the *t*-plane, where *t* = *r* + *is*. (**f**) The deformed field lines and equipotential lines in the *z*-plane.

**Figure 5:**
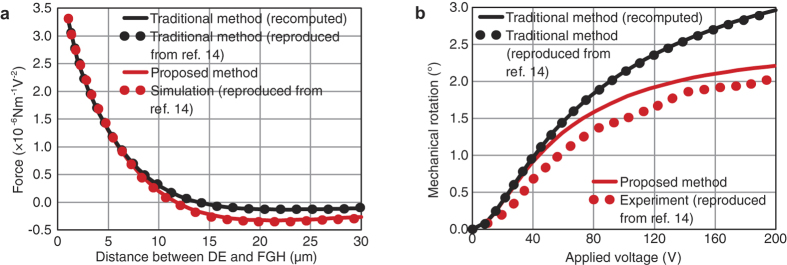
Theoretical, simulation and experimental results of Fig. 4. (a) Comparisons of the electrostatic force obtained in ref. 14 and in this study. Our results based on the proposed conformal mapping method are more consistent with the simulation results in ref. 14 than those based on the traditional method. (**b**) Comparisons of the rotation angle obtained in ref. 14 and in this study. Compared with the theoretical results based on the traditional method, the theoretical results based on the proposed method are considerably closer to the experimental results of ref. 14.

In 2003, He *et al*. proposed an out-of-plane electrostatic repulsive actuator^36^, which has been widely used in translation and rotation micromirrors^37^,^38^. Figure 4a shows a schematic diagram of the actuator. It is a periodic arrangement of three electrodes, namely, an unaligned fixed electrode, an aligned fixed electrode, and a moving electrode. When the unaligned fixed electrodes are grounded, with a voltage *V* applied to both the aligned fixed electrodes and the moving electrodes, the moving electrodes will be driven vertically upward as if they are repulsed by the fixed electrodes. By calculating the electrostatic force applied to the moving electrodes as a function of their heights, the performances of the electrostatic repulsive actuators can be obtained. Ref. 14 studied this actuator thoroughly and Fig. 4b shows the modeled structure. Solid edges BC, DE, and FGH denote an unaligned fixed electrode, an aligned fixed electrode, and a moving electrode, respectively; dashed edges denote electric field lines. In ref. 14, as indicated in Fig. 4c, by extending electrodes DE and FGH to infinity, the two electrodes were approximated by one electrode DEFG, and then the traditional conformal mapping method was employed. The analytical results are reproduced (from Fig. 6 of ref. 14) as a black dotted line in Fig. 5a. To evaluate the accuracy of the method, the authors also provided simulation results produced by a numerical simulation software, which are reproduced (also from Fig. 6 of ref. 14) as a red dotted line in Fig. 5a.

We repeated their analytical calculations using the traditional conformal mapping method with the parameter values provided by Fig. 5 of ref. 14; the results are shown as a black solid line in Fig. 5a. As expected, our results agree well with those in ref. 14. According to the comparison between the analytical and simulation results, when the distance between the conductors DE and FGH is small, the two results are in agreement. However, when the distance becomes large, the agreement worsens. This discrepancy was mainly because of the fact that conductors DE and FGH were approximated as a single conductor; this was explained in ref. 14 and will be verified later.

We recalculated the structure shown in Fig. 4b using the proposed conformal mapping method. The structure is a generalized polygon. A complex plane, (the *z*-plane), is constructed such that vertex B is the origin and BE is the real axis. Thus, the coordinates of all vertices in the *z*-plane, namely, *z*_A_, *z*_B_, *z*_C_, *z*_D_, *z*_E_, *z*_F_, *z*_G_, and *z*_H_, can be derived on the basis of the structural parameters of the electrodes. Through inverse Schwarz–Christoffel (SC) mapping^39^, Fig. 4b can be mapped onto the upper half-plane of another complex plane, the *w*-plane, as shown in Fig. 4d. The coordinates of any three vertices in the *w*-plane can be chosen arbitrarily, e.g., −1 (vertex B), 1 (vertex H), and infinity (vertex A). The mapping function from the *w*-plane to the *z*-plane^39^ is:


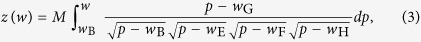


where *w*_B_, …, *w*_H_ are the complex coordinates of the corresponding vertices in the *w*-plane and *M* is a complex constant. Except for the three chosen coordinates (*w*_B_, *w*_H_, and *w*_A_), the remaining coordinates and *M* are unknown and can be obtained by solving a system of nonlinear equations, *z*_A_ = *z*(*w*_A_), *z*_B_ = *z*(*w*_B_), …, *z*_H_ = *z*(*w*_H_). Because of the lack of any analytical solution for these coordinates, they are solved numerically with the SC toolbox of MATLAB created by T. A. Driscoll^40^,^41^.

Figure 4e shows that the upper half-plane of the *w*-plane can be further mapped onto a folded rectangle in another complex plane, the *t*-plane, via forward SC mapping. The mapping function is


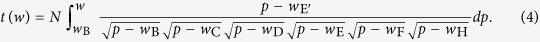


In the above function, *w*_E’_ is unknown and should be solved before the mapping is performed. As analyzed in the previous section, in the *t*-plane the length EE’ should be equal to the length E’F, i.e., the length EF is zero. Since *t*_F_ − *t*_E_ = 0 = *t*(*w*_F_) − *t*(*w*_E_), *w*_E’_ should satisfy the following:


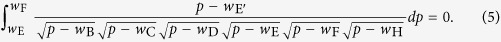


Thus,


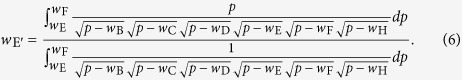


After solving for *w*_E’_, equation (4) can be solved numerically in MATLAB. With the two mapping functions, equation (3) and equation (4), the electric field distribution in Fig. 4b will be obtained. Figure 4f shows the computed electric field lines and equipotential lines. The electrostatic force can be computed by using the same method as that used in ref. 14. The calculated force is plotted as a red solid line in Fig. 5a. Compared with the analytical results of ref. 14, the calculation results based on the proposed conformal mapping method were more consistent with the simulation results of ref. 14. This observation shows that the approximation of three electrodes to two electrodes is the main reason for the discrepancy between the black dotted line and the red dotted line in Fig. 5.

On the basis of the electrostatic repulsive actuator discussed above, a rotation micromirror was developed in ref. 14. The theoretical and experimental performances (rotation angle versus applied voltage) achieved in ref. 14 are reproduced (from Fig. 16 of ref. 14) in Fig. 5b as a black dotted line and a red dotted line, respectively. On the basis of our calculated results of electrostatic force, we then recalculated the rotation angle. The results of the traditional mapping method based on the approximate structure (Fig. 4c) and the results of the proposed mapping method based on the accurate structure (Fig. 4b) are also shown as a black solid line and a red solid line, respectively, in Fig. 5b. Compared with the theoretical values given in ref. 14, our theoretical values based on the proposed method are considerably closer to the experimental values given in ref. 14. This outcome shows the effectiveness and accuracy of the proposed method.

### Transformation optics splitter

**Figure 6:**
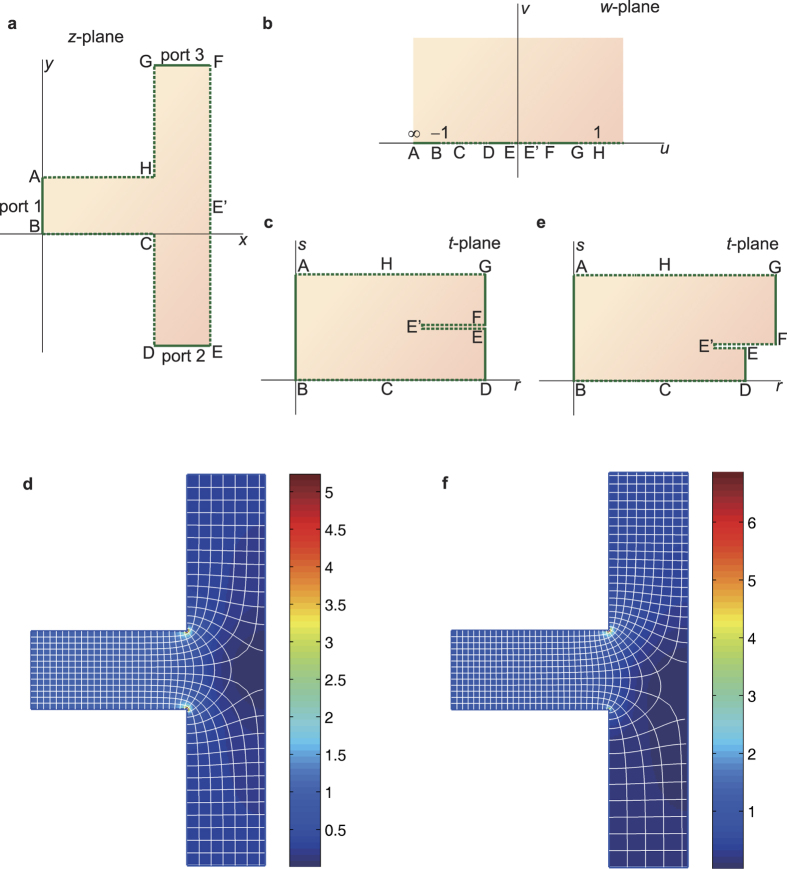
Conformal mapping processes for a beam splitter with three ports. (**a**) Structure of the beam splitter. The three solid lines represent the three ports for input or output. (**b**) Splitter mapped onto the upper half-plane of the *w*-plane. (**c**) Final mapped structure of the splitter when the incoming beam is equally split into two beams. (**d**) The permittivity distribution and the curved coordinate grid in the splitter when it is mapped onto (**c**). (**e**) Final mapped structure of the splitter when the incoming beam is unequally split into two beams. (**f**) The permittivity distribution and the curved coordinate grid in the splitter when it is mapped onto **e**. The split ratio of port 2 to port 3 is 1/2.

Refractive index *n*, or relative permittivity *ε*_*r*_ and relative permeability *μ*_*r*_ (*n*^2^ = *ε*_*r*_*μ*_*r*_), are key parameters that affect the propagation, reflection, and refraction of electromagnetic waves in a region or medium. In transformation optics, through coordinate transformation (including conformal transformation), a complicated region with a special refractive index distribution can be transformed to a simple region with a special refractive index distribution (usually a uniform distribution). The transformation between regions is associated with the transformation between index distributions. With this method, various structures or devices with complicated index distributions can be designed to control electromagnetic fields. In ref. 19 a T-shaped light beam splitter was designed, as described in Fig. 6a. AB (port 1), DE (port 2) and FG (port 3) are the three ports of the splitter. By carefully designing the refractive index distribution in the shaded region, the incoming beam through port 1 is equally split into two beams, which then smoothly pass through the 90° and −90° bends and finally exit through ports 2 and 3, respectively. In the literature, the splitter was designed by solving Laplace’s equations numerically. The maximum index was observed near the vertices C and H and was about 6.7 times the index in port 1. The minimum index was observed near the midpoint of EF.

As demonstrated in the calculations of the electrostatic actuator, we construct a *z*-plane, as shown in Fig. 6a. An intermediate geometry, e.g., the upper half-plane of the *w*-plane (Fig. 6b), is still used. Similarly, the mapping function from the *w*-plane to the *z*-plane is


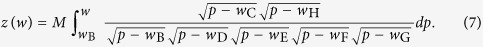


Next, given the design goal, the mapped splitter in the *t*-plane should resemble that shown in Fig. 6c. Considering the symmetry, the point E’ in the *z*-plane should be the midpoint of EF. The mapping function from the *w*-plane to the *t*-plane is


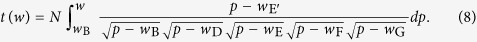


The refractive indices of the shaded region in the *z*-plane, *w*-plane, and *t*-plane are denoted as *n*_*z*_, *n*_*w*_, and *n*_*t*_, respectively. They are determined by the following equations^17^:


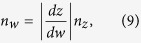



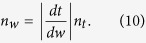


Taking derivatives of equations (7) and (8) with respect to *w*, substituting them into equations (9) and (10), respectively, and assuming that the refractive index in the *t*-plane is 1 (vacuum), the index in the *z*-plane can be expressed as


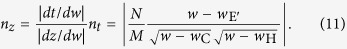


So, the index *n*_*z*_ is determined by a relatively simple formula, equation (11). Some specific values of *n*_*z*_ can be easily determined with this equation: (1) *n*_*z*_ will equal *N*/*M* when *w* is infinity. In the *w*-plane, the coordinate of vertex A is infinity. Therefore, the complex constants *M* and *N* are set to be equal to ensure the index of vertex A (the entrance of the splitter) is equal to 1; (2) By assuming that the denominator and numerator of equation (11) are equal to zero, the maximum and minimum values of *n*_*z*_ (infinity at vertices C and H and zero at vertex E’, respectively) can be obtained. However, it is difficult or even impossible to calculate these extremum values for method described in ref. 19. To enable comparison of ref. 19 with equation (11), calculated results of the distribution of relative permittivity *ε*_*r*_ = *n*_*z*_^2^ are shown in Fig. 6d. Through inverse mapping, the calculated curved coordinate grid is also shown in Fig. 6d.

The results shown in ref. 19 rely on a particular symmetry; the shapes and sizes of port 2 and port 3 must be the same, the positions of port 2 and port 3 must be symmetric, and the amplitudes and phases of emergent light in port 2 and port 3 must be equal. However, our proposed method is applicable to an arbitrary configuration. For example, to split the incoming beam into two unequal beams, the structure shown in Fig. 6a should be mapped onto an asymmetric structure, as shown in Fig. 6e. Now E’ is not the midpoint of EF and its position can be calculated through the split ratio *SR* = *L*_DE_/*L*_FG_ in the *t*-plane, where *L*_DE_ and *L*_FG_ is the length of port 2 and port 3, respectively. The mapping functions and refractive index are still determined by equations (7), (8) and (11). The computed permittivity distribution and curved coordinate grid when *SR* = 1/2 are shown in Fig. 6f. Two thirds of the incoming beam exits through port 3 while the other one third exits through port 2.

In conclusion, to overcome the limitations of traditional conformal mapping and extend its application from two terminals to multiple terminals, a novel conformal mapping method was proposed through folding the additional terminals or boundaries into the inner part of the mapped structure. Two application examples of the proposed method are shown and the results were compared with the literature. From the comparison of the results of the two examples, the proposed method is more accurate without increasing complexity. So, the proposed method may enable more accurate design of complicated devices in a variety of research fields.

## Additional Information

**How to cite this article**: Wang, W. *et al*. Conformal mapping for multiple terminals. *Sci. Rep*. **6**, 36918; doi: 10.1038/srep36918 (2016).

**Publisher’s note:** Springer Nature remains neutral with regard to jurisdictional claims in published maps and institutional affiliations.
